# Evaporation-induced hydrodynamics control plasmid transfer during surface-associated microbial growth

**DOI:** 10.1038/s41522-023-00428-x

**Published:** 2023-08-22

**Authors:** Chujin Ruan, Benedict Borer, Josep Ramoneda, Gang Wang, David R. Johnson

**Affiliations:** 1 College of Land Science and Technology, China Agricultural University, Beijing, China; 2 Department of Environmental Microbiology, Swiss Federal Institute of Aquatic Science and Technology (Eawag), Dübendorf, Switzerland; 3 Department of Earth, Atmospheric and Planetary Sciences, Massachusetts Institute of Technology, Cambridge, MA USA; 4 Cooperative Institute for Research in Environmental Sciences, University of Colorado, Boulder, CO USA; 5 National Black Soil & Agriculture Research, China Agricultural University, Beijing, China; 6 Institute of Ecology and Evolution, University of Bern, Bern, Switzerland

**Keywords:** Biofilms, Antimicrobials

## Abstract

Droplet evaporation is a general process in unsaturated environments that results in micro-scale hydrodynamic flows which in turn determine the spatial distributions of microbial cells across surfaces. These spatial distributions can have significant effects on the development and functioning of surface-associated microbial communities, with consequences for important processes such as the spread of plasmids. Here, we experimentally quantified how evaporation-induced hydrodynamic processes modulate the initial deposition patterns of microbial cells (via the coffee ring effect and Marangoni convection) and how these patterns control the spread of an antibiotic resistance-encoding plasmid during surface-associated growth. We found that plasmid spread is a function of the initial density of cells deposited along the droplet periphery, which is a manifestation of the coffee ring effect. Using an individual-based model, we systematically linked how the different initial cell deposition patterns caused by the relative strengths of the coffee ring effect and Marangoni convection determine the extent of plasmid transfer during surface-associated growth. Our study demonstrates that evaporation-induced hydrodynamic processes that are common in nature can alter crucial ecological properties of surface-associated microbial communities and control the proliferation of plasmids, with consequences on the spread of antibiotic resistance and other plasmid-encoded traits.

## Introduction

Surface-associated microbial lifestyles (e.g. biofilms, colonies, bioaggregates, etc.) are omnipresent in both natural and engineered environments^1–4^ and have important roles in ecosystem functioning^5^. Such lifestyles begin with the deposition (either active or passive) of microbial cells onto a surface, followed by their growth and development into complex sessile communities^6–8^. The initial deposition pattern of cells across a surface can determine the direction of microbial community development, as different deposition patterns can lead to the formation of distinct architectures, alter microbial interactions, and determine the spatial positionings of different cells^9^. These spatial features, in turn, can dictate crucial processes such as nutrient acquisition and community productivity^10,11^, the exchange of intermediate metabolites^12–14^, horizontal gene transfer^15–18^, and resistance to phage infection^19^, among others. Therefore, understanding the factors that influence cell deposition and subsequent surface-associated growth can provide crucial insights into the functioning and behaviors of microbial communities and their impact on the environment. Moreover, this knowledge can aid in the development of strategies to manipulate such communities for beneficial purposes.

Plasmid transfer among microbes can impact their ecology and evolution by conferring adaptive traits, particularly in the context of biofilms and microbiomes^17,18,20–22^. Importantly, plasmid-encoded traits have fundamental roles in major problems facing our society and environment, such as antibiotic resistance and xenobiotic degradation^20–23^. Numerous studies have identified environmental factors that affect plasmid transfer and fate^24–26^, including temperature, pH, humidity, and nutrient concentrations, which have unquestionably improved our ability to predict plasmid dynamics in the environment^20,21^. However, these studies often neglect (or only indirectly consider) the fact that plasmid transfer typically requires direct contact between a plasmid donor and a potential recipient cell. The deposition pattern of cells across a surface, which determines the number of contacts between plasmid donor and potential recipient cells, is therefore an important determinant of plasmid transfer and fate, especially in systems where cells have limited dispersal capabilities such as in soils^27^ and engineered biofilm systems^22^.

Evaporation, which is an inevitable process in unsaturated environments, can have profound effects on the deposition pattern of cells, and thus on the number of contacts between plasmid donor and potential recipient cells^28^. Many microbes are dispersed via water droplets (e.g. rainfall, aerosols from wastewater treatment plants and surface waters, etc.) that enable them to traverse long distances and cross geographic barriers^29–31^. Upon contact of a droplet with a surface, evaporation will cause micro-scale liquid convection within the droplet as a function of surface wettability^32^, which can determine the initial deposition pattern of cells across the surface, the number of contacts between plasmid donor and potential recipient cells, and the number of plasmid transfer events. Despite numerous studies investigating the environmental factors that affect plasmid transfer^24,25^, the influence of the initial cell deposition pattern as determined by evaporation-induced hydrodynamics on plasmid transfer and fate remains understudied.

**Fig. 1:**
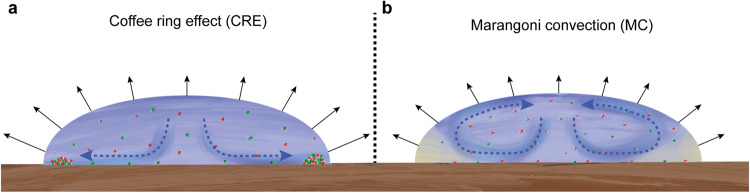
Conceptual depiction of cell deposition under different evaporation-induced hydrodynamics. **a** The coffee ring effect (CRE) is caused by preferential evaporation along the surface-pinned liquid-air interface (the contact line). This causes capillary flows toward the contact line and the accumulation of cells at the droplet periphery. **b** Marangoni convection (MC) is caused by temperature gradients or surfactants that reduce the differential effect of evaporation across the liquid-air interface due to the accumulation of the surfactant at the contact line (illustrated here as the yellow gradient). This causes the formation of circular vortices that redistribute cells and results in a more homogeneous cell deposition pattern.

Evaporation of a particle (cell)-laden droplet often leads to a highly heterogeneous deposition pattern termed the “coffee ring effect” (hereafter referred to as the CRE) (Fig. 1a)^32,33^. The CRE occurs when liquid preferentially evaporates along the surface-pinned liquid-air interface (the contact line) and is replenished from the center of the droplet, resulting in a net transport of suspended particles (cells) from the center to the periphery of the droplet (Fig. 1a). Evaporation of a droplet can also induce a convective Marangoni flow due to a gradient in surface tension (Fig. 1b). This is caused by temperature gradients resulting from preferential evaporation along the contact line (thermal Marangoni flow)^34^, but can also be caused by surfactants that accumulate at the contact line and locally lower the surface tension (surfactant induced Marangoni flows) (Fig. 1b)^35^. These convective Marangoni flows (hereafter referred to as MC) counteract the above-mentioned net transport of particles (cells) to the contact line by the CRE^36^, and the deposition pattern is thus a function of the relative strengths of the CRE and MC (Fig. 1). These two effects are not mutually exclusive and both can occur at the same time depending on the solute potential, the production or presence of surfactants, and the surface wettability^35,36^.

To test the hypothesis that evaporation-induced hydrodynamic processes determine the cell deposition pattern and the number of contacts between plasmid donor and potential recipient cells, and thus the extent of plasmid transfer during surface-associated growth, we experimentally manipulated and quantified the effects of the CRE and MC. During evaporation of the droplet, we expected the CRE to concentrate cells at the droplet periphery (i.e., the contact line) whereas MC would counteract these effects and result in a more homogeneous cell deposition pattern across the entire surface. We further expected that the increased concentration of cells at the droplet periphery due to the CRE would increase the number of contacts between plasmid donor and potential recipient cells and increase the extent of plasmid transfer during surface-associated growth. To test these expectations, we performed experiments where we allowed two fluorescently-labeled strains of the bacterium *Escherichia coli* to grow and spread across nutrient-amended agar surfaces. These strains can engage in conjugation-mediated transfer of the plasmid R388, which encodes for a fluorescent protein and antibiotic resistance. While agar surfaces do not mimic solid abiotic surfaces such as soil particles, they do mimic biotic surfaces such as skin, leaves, and roots in the sense that they contain water and supply nutrients. We controlled the strength of MC by adding a biologically inert surfactant to the cell-laden droplet, where increasing the surfactant intensifies MC^34,35,37^. We then quantified the extent of plasmid transfer as a function of the evaporation-induced hydrodynamics using confocal laser-scanning microscopy (CLSM) and quantitative image analysis. Finally, we used an individual-based computational model to identify mechanisms by which evaporation and the initial cell deposition pattern can regulate plasmid transfer during surface-associated growth.

## Results

### Cell deposition dominated by the coffee ring effect increases the number of cell contacts

We first tested how different evaporation-induced hydrodynamic processes (CRE or MC) affect the number of cell contacts between different strains during surface-associated growth. To achieve this, we performed surface-associated growth experiments with *E. coli* strains TB204 and TB205, which constitutively express green or red fluorescent protein from the chromosome, respectively, but are otherwise genetically and phenotypically identical. We controlled the evaporation-induced hydrodynamic flows within the inoculation droplet by altering the relative strengths of the CRE and MC by adding the biologically-inert surfactant polyethylene glycol (Supplementary Fig. 1a). During the experiment, we quantified two proxies for the number of cell contacts between different strains. The first is the number of discrete sectors of each strain that form during surface-associated growth, where more sectors correspond to more interfaces (and thus more cell contacts) between the two strains. Briefly, only a few cells positioned at the biomass periphery have access to resources supplied from the periphery and are actively growing, which causes different strains to segregate into different local patches (i.e. sectors) as a consequence of stochastic drift along the biomass periphery^38^. The second is the intermixing index *I*_*r*_, which measures the number of strain (color) transitions normalized by the expected number of transitions for a random spatial distribution of two strains at a certain radial distance r from the biomass centroid. The intermixing index *I*_*r*_ is calculated as *I*_*r*_ = 2*N*_*r*_/*πr*, where *r* is the radial distance from the biomass centroid and *N*_*r*_ is the number of strain transitions at the distance r from the biomass centroid (i.e., *I*_*r*_ can be calculated for any distance from the biomass centroid).

**Fig. 2:**
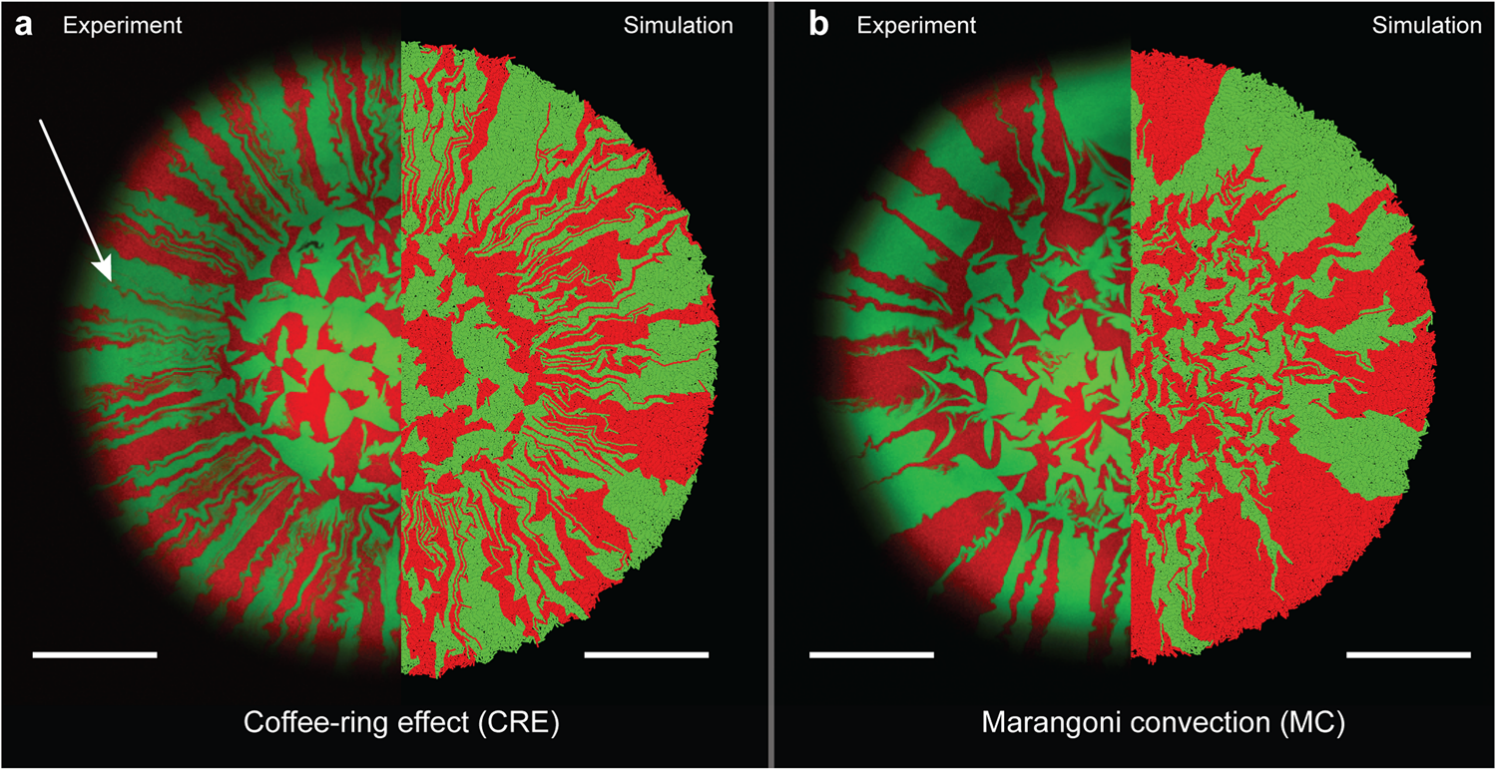
Spatial patterns that formed during surface-associated growth after cell deposition under different evaporation-induced hydrodynamics. Images are for cell deposition dominated by **a** the coffee-ring effect (CRE) or **b** Marangoni convection (MC). For both panels, the experiments are the left hemispheres and the simulations are the right hemispheres. For the experiments, the images are representative CLSM images at the end of the experiment. Strain TB204 expressed green fluorescent protein and strain TB205 expressed red fluorescent protein. Neither strain carried plasmid R388, and the strains were therefore phenotypically and genetically identical other than expressing different fluorescent proteins. The white arrow indicates a boundary coalescence event. The scale bars for the experiments are 1000 µm and for the simulations are 100 µm.

We found that a larger number of sectors formed during surface-associated growth when cell deposition was dominated by the CRE (mean = 105, SD = 12.5, *n* = 5) (Fig. 2a) than by MC (mean = 48, SD = 4.42, *n* = 5) (Fig. 2b) (two-sample two-sided Welch test; *p* = 2.1 × 10^−4^, *n* = 5). Dominance of the CRE resulted in numerous sectors emerging from the initial droplet area that were rapidly lost through boundary coalescence events (spatial bottlenecks) during surface-associated growth (Fig. 2a; white arrow). In contrast, dominance of MC resulted in relatively few sectors emerging from the initial droplet area that mostly persisted to the final biomass periphery with only a few exceptions (Fig. 2b). Our individual-based computational simulations show congruent results, where we also observed a larger number of sectors persisting during surface-associated growth when cell deposition was dominated by the CRE (mean = 89, SD = 5.45, *n* = 5) (Fig. 2a) than by MC (mean = 34, SD = 10.6, *n* = 5) (Fig. 2b) (two-sample two-sided Welch test; *p* = 7.2 × 10^−5^, *n* = 5). Thus, increasing the relative strength of the CRE resulted in more sectors, and thus more cell contacts, between the two strains.

**Fig. 3:**
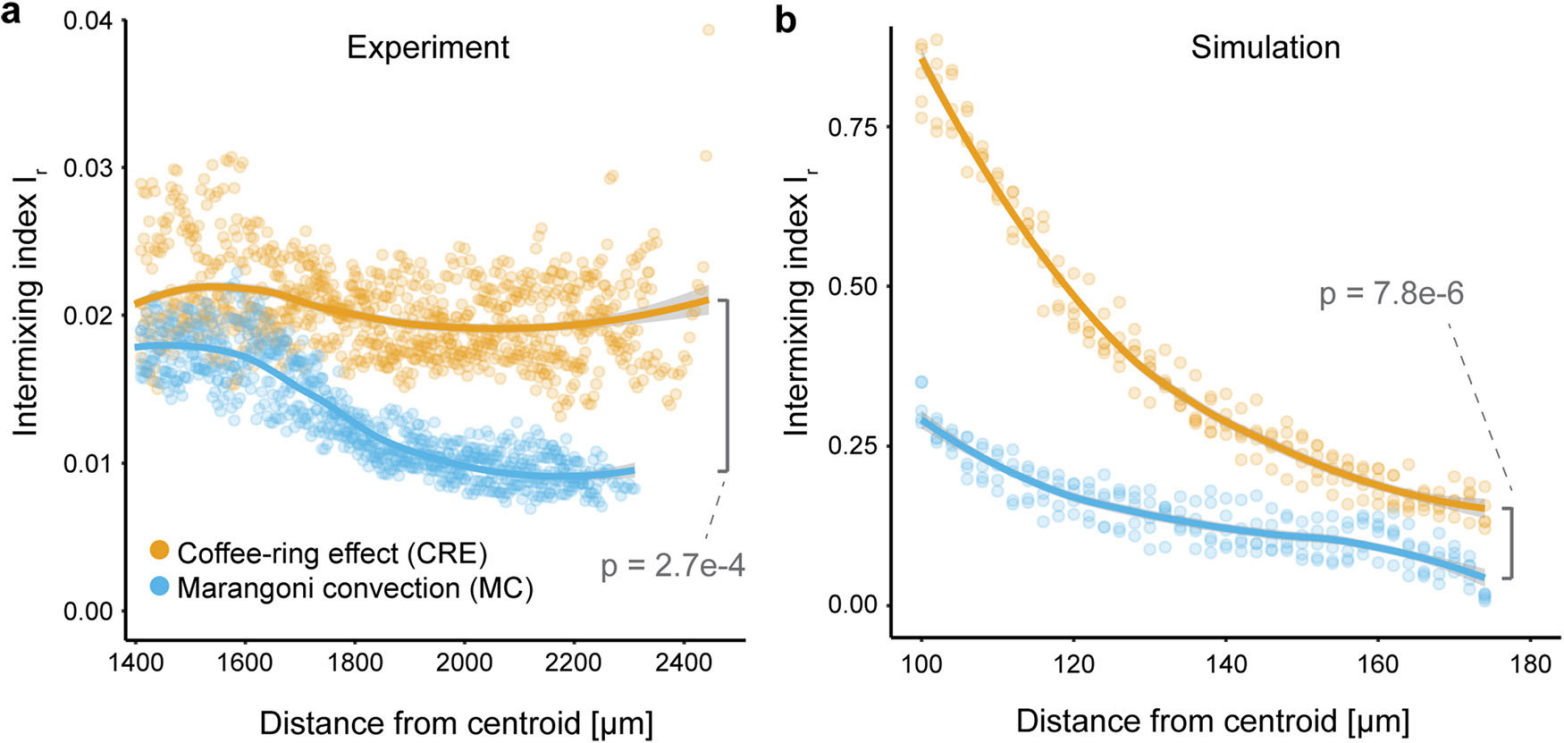
Dynamics of spatial intermixing during surface-associated growth after cell deposition under different evaporation-induced hydrodynamics. **a** Intermixing index as a function of the radial distance from the centroid of the biomass for the experiments. Note that the initial droplet had a radius of approximately 1400 µm, which is the radius at which biomass growth began. **b** Intermixing index as a function of the radial distance from the centroid of the biomass for the simulations. Note that the initial droplet had a radius of approximately 100 µm, which is the radius at which biomass growth began. For the experiments, the quantities were obtained from CLSM images at the end of the experiment with strains TB204 and TB205. For all panels, each datapoint is a measurement for one of five replicates and the solid lines are running averages. The *p*-values are for two-sample two-sided Welch tests. Statistical differences are calculated for the means of the intermixing indices for a band with a width of 35 µm located at the biomass periphery.

Congruent with our analyses of sector formation, cell deposition dominated by the CRE resulted in higher spatial intermixing indices when compared to cell deposition dominated by MC (calculated at the biomass periphery; two-sample two-sided Welch test; *p* = 2.1 × 10^−4^, *n* = 5) (Fig. 3). Interestingly, cell deposition dominated by the CRE did not lead to a significant decrease in the intermixing index in the experiments (two-sided Pearson correlation test; *r* = −0.027, *p* = 0.37, *n* = 5) (Fig. 3a). In contrast, the intermixing index gradually declined when MC was dominant, which is in line with the simulations (two-sided Pearson correlation test; *r* = -0.83, *p* = 2.2 × 10^−16^, *n* = 5) (Fig. 3 and Supplementary Movies 1 and 2). The simulations also predicted a rapid decline in the intermixing index when the CRE was dominant that we did not observe in our experiments (Fig. 3). Overall, when comparing the intermixing indices at the biomass periphery (defined as a 35 μm-wide band located at the biomass periphery^11^), dominance of the CRE resulted in a significantly higher intermixing index when compared to dominance of MC in both experiments (two-sample two-sided Welch test; *p* = 2.7 × 10^−4^, *n* = 5) (Fig. 3a) and simulations (two-sample two-sided Welch test; *p* = 7.3 × 10^−8^, *n* = 5) (Fig. 3b).

### Cell deposition dominated by the coffee ring effect leads to increased transfer of plasmid R388

**Fig. 4:**
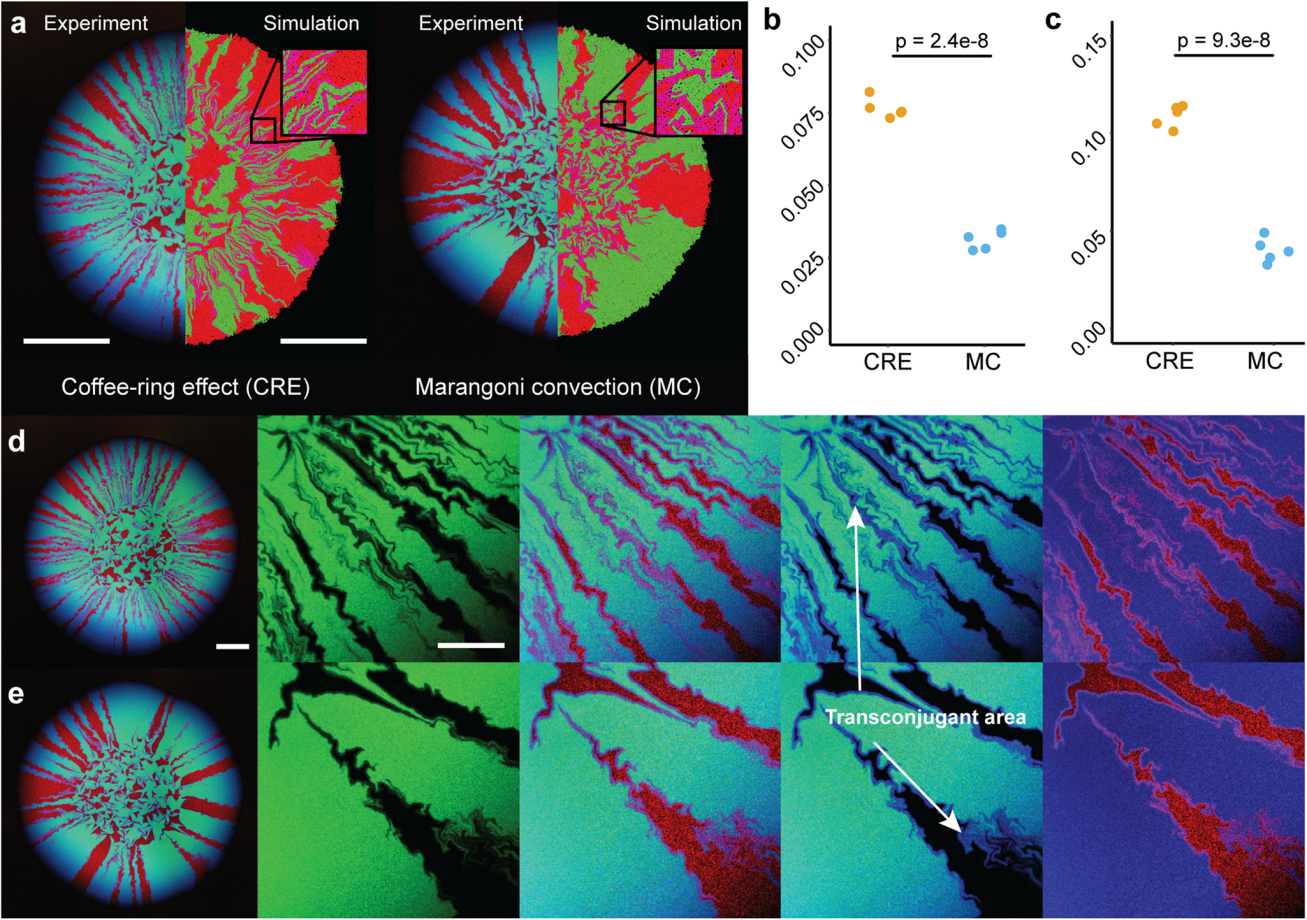
Transfer of plasmid R388 during surface-associated growth after cell deposition under different evaporation-induced hydrodynamics. **a** Images are for cell deposition dominated by the coffee-ring effect (CRE) or Marangoni convection (MC). Experimental results are the left hemispheres and the simulations are the right hemispheres. For the experiments, the images are representative CLSM images at the end of the experiment. Strain TB205 (potential recipient strain) expresses red fluorescent protein from the chromosome while strain TB206 (R388 donor strain) expresses both green fluorescent protein from the chromosome and cyan fluorescent protein from R388. If strain TB205 successfully receives R388, it expresses both red and cyan fluorescent protein and appears as the composite color magenta. The scale bars for the experiments are 1000 µm and for the simulations are 100 µm. **b** Quantification of the transconjugant area when cell deposition was dominated by the CRE or MC in the experiments. **c** Quantification of the transconjugant area when cell deposition was dominated by the CRE or MC in the simulations. For **b** and **c**, each datapoint is a measurement for one of five replicates and the *p*-values are for two-sample two-sided Welch tests. Images of successful R388 transfer in the experiments when cell deposition was dominated by the **d** CRE or **e** MC. For **d**, **e**, the images show (from left to right) the entire biomass area, an enlarged view of the highlighted rectangle in the left image with only the green channel to identify the R388 donors (expressing green fluorescent protein) (second image), the enlarged view with all fluorescence channels (third image), the enlarged region with the red channel removed to highlight transconjugants (expressing cyan but not green fluorescent protein) (fourth image), and the enlarged region with the green channel removed to highlight the R388 donors (expressing only cyan fluorescent protein), potential recipients (expressing only red fluorescent protein), and transconjugants (expressing both cyan and red fluorescent protein). Note that transconjugants only emerge along the interfaces between strains TB205 (potential recipient strain) and TB206 (R388 donor strain). The scale bars are 1000 µm for the entire expansions and 400 µm for the enlarged regions.

Because the relative strengths of the CRE and MC strongly influenced sector formation and spatial intermixing during surface-associated growth, we hypothesized that the initial cell deposition patterns controlled by the CRE or MC would also affect the extent of plasmid transfer during surface-associated growth. This is because the initial cell deposition pattern determines the number of cell contacts between different strains (extent of sector formation and spatial intermixing), and thus the number of possible plasmid transfer events. To test this hypothesis, we used strain TB206 as a plasmid donor strain, which constitutively expresses green fluorescent protein from the chromosome and cyan fluorescent protein and chloramphenicol resistance from the conjugative plasmid R388. Strain TB206 is otherwise identical to strain TB204. In combination with the potential recipient strain TB205, which constitutively expresses red fluorescent protein from the chromosome, we were able to identify and track successful R388 transfer events by combining the different fluorescence information (Fig. 4). Because contact between an R388 donor and a potential recipient cell is required for successful R388 transfer, we expected transconjugant cells to emerge and proliferate along the interfaces of the R388 donor and potential recipient sectors.

As expected, we observed higher relative transconjugant areas when the CRE was dominant than when MC was dominant in both experiments (two-sample two-sided Welch test; *p* = 2.4 × 10^−8^, *n* = 5) (Fig. 4a, b) and simulations (two-sample two-sided Welch test; *p* = 9.3 × 10^−8^, *n* = 5) (Fig. 4a, c and Supplementary Movies 3 and 4). For both the experiment and simulations, we calculated the relative transconjugant area as the area occupied by transconjugant cells divided by the total area of the biomass (i.e., we considered both the inoculation area and the expansion area). For the simulations, we implemented a 10% reduction in the growth rate when carrying a plasmid, which is congruent with our experiments (Supplementary Fig. 1b). To further validate this result, we quantified the relative number of transconjugant cells using conventional colony counting on chloramphenicol-amended LB agar plates (Supplementary Fig. 1c). These results confirm that the population-level extent of R388 transfer was significantly higher when cell deposition was dominated by the CRE (mean = 7.6%, SD = 0.3%, *n* = 5) than by MC (mean = 3.1%, SD = 0.3%, *n* = 5) (two-sample two-sided Welch test; *p* = 1.8 × 10^−8^, *n* = 5).

### Cell deposition dominated by the coffee ring effect increases plasmid transfer by altering the local cell density at the droplet periphery

**Fig. 5:**
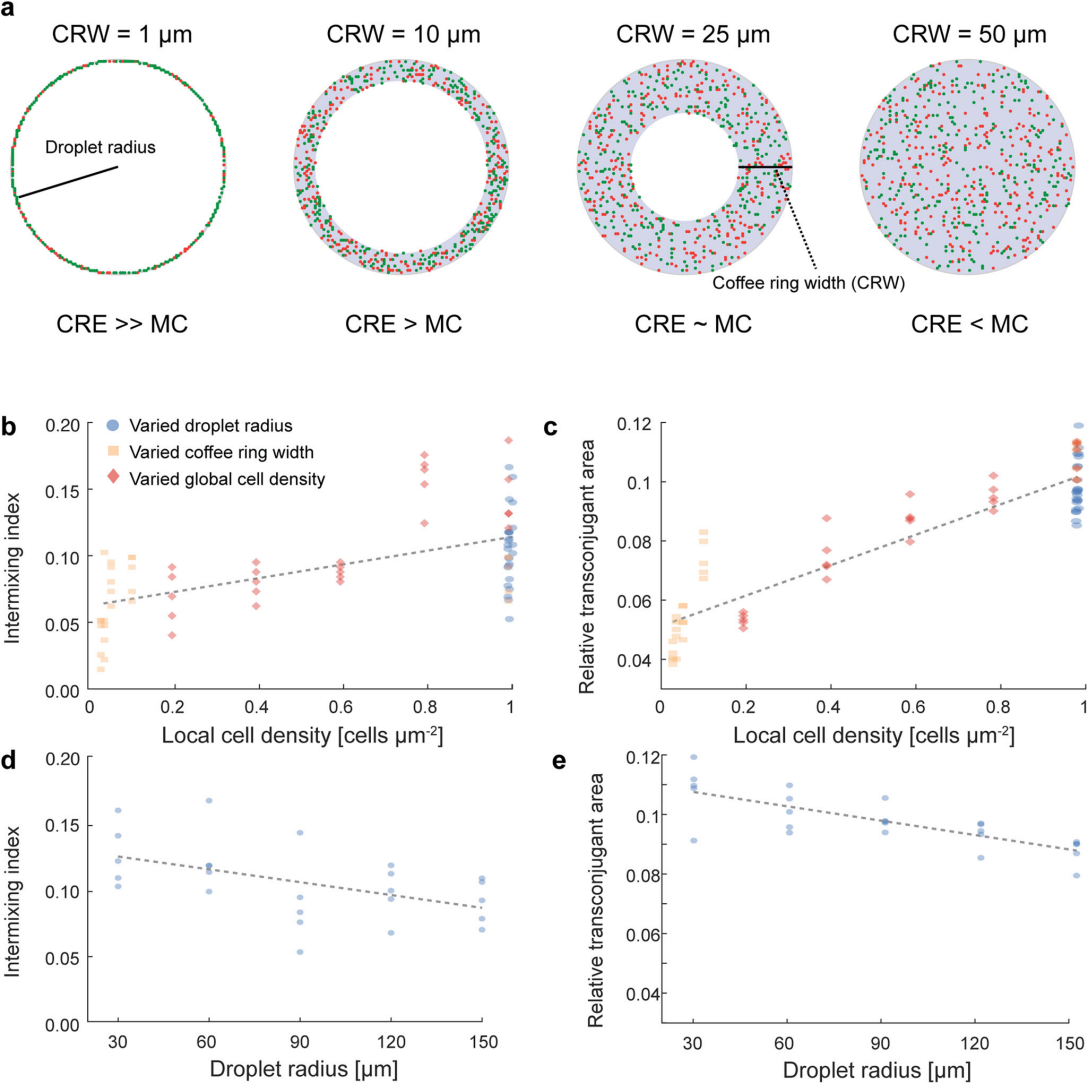
Intermixing index and transconjugant area as a function of the local cell density at the initial droplet periphery and the initial droplet radius. We quantified these relationships from the individual-bases simulations. **a** Visualization of how the coffee ring width alters the local cell density for equal numbers of inoculated cells. **b** Quantification of the intermixing index at the final biomass periphery as a function of the local cell density at the initial droplet periphery. **c** Relative transconjugant area after surface-associated growth as a function of the local cell density in the inoculation area. **d** Quantification of the intermixing index at the final biomass periphery after surface-associated growth as a function of the initial droplet radius but adjusting the cell number to ensure a constant local cell density. **e** Relative transconjugant area after surface-associated growth as a function of the initial droplet radius but adjusting the cell number to ensure a constant local cell density. The local cell density here is calculated from geometrical considerations and the inoculated cell number across the explored parameter space of the simulations (Supplementary Figs. 2-4). The local cell density serves as a proxy for the relative strengths of the CRE and MC. For **b**–**e** each data point is a measurement for one of five independent simulations and the lines are linear regression model fits to the data (statistics provided in Supplementary Table 2).

We finally sought to understand how cell deposition by the CRE and MC cause differences in spatial intermixing and plasmid transfer during surface-associated growth by finely controlling the initial cell deposition pattern using our individual-based simulations. We imposed dominance of the CRE by randomly distributing 628 cells within a ring with a width of 1 µm and an outer radius of 100 µm (fully packed ring with a width of approximately one cell length). In these simulations, the outer diameter of the ring represents the diameter of the simulated droplet. We then changed the ring width (penetration distance into the simulated droplet), which allowed us to simulate different relative strengths of the CRE and MC (Fig. 5a). If the ring width equals the initial droplet radius, the system is dominated by MC and the local cell density at the droplet periphery is relatively small. In contrast, if the ring width is small compared to the initial droplet radius, the system is dominated by the CRE and the local cell density at the droplet periphery is large. Using this approach, we systematically varied the total number of inoculated cells (Supplementary Fig. 2), the simulated initial droplet radius (Supplementary Fig. 3), and the ring width (Supplementary Fig. 4) and calculated the intermixing index at the biomass periphery and the transconjugant area as a function of the local cell density. We thus used the local cell density as a proxy for the relative strength of the CRE and MC.

Using this approach, we found significant correlations between the intermixing index at the final biomass periphery and the local cell density when varying the total number of inoculated cells (two-sided Pearson correlation test; *r* = 0.59, *p* = 2.9 × 10^−8^, *n* = 5), the simulated initial droplet radius (two-sided Pearson correlation test; *r* = −0.58, *p* = 6.7 × 10^−8^, *n* = 5), and the ring width (two-sided Pearson correlation test; *r* = −0.56, *p* = 1.7 × 10^−7^, *n* = 5) (Fig. 5b and Supplementary Figs. 2-4). We also found significant correlations between the relative transconjugant area and the local cell density when varying the total number of inoculated cells (two-sided Pearson correlation test; *r* = 0.65, *p* = 4.3 × 10^−10^, *n* = 5), the simulated initial droplet radius (two-sided Pearson correlation test; *r* = −0.604, *p* = 9.6 × 10^−9^, *n* = 5), and the ring width (two-sided Pearson correlation test; *r* = −0.55, *p* = 3.6 × 10^−7^, *n* = 5) (Fig. 5c and Supplementary Figs. 2-4). Thus, the relative strengths of the CRE and MC have profound effects on spatial intermixing and plasmid transfer during surface-associated growth by modulating the local cell density at the growing biomass periphery.

When varying the initial droplet radius in the simulations described above, we adjusted the number of deposited cells such that the local cell density always remained 1. However, varying the initial droplet radius will change the strength of competition for unoccupied space ahead of the deposited cells (Supplementary Fig. 5). The sector width available to place kin cells into unoccupied space is related to the droplet radius as *α*=2*π*/2*rπ*=1/*r*, with the assumption that one cell is positioned at each micron along the initial droplet circumference (congruent with the simulations, see Supplementary Table 1 for a summary of all initial conditions in the simulations). Interestingly, despite having the same local cell density at the initial droplet periphery, we observed a decrease in both the intermixing index at the final biomass periphery (Fig. 5d) and the relative transconjugant area (Fig. 5e) as the initial droplet radius increased.

## Discussion

Evaporation is an important driver of cell and nutrient distributions on hydrated surfaces whose consequences for environmentally-relevant functions such as antibiotic resistance spread remain understudied. Here, we quantified how evaporation-induced hydrodynamic processes affect the spatial distribution patterns of cells and the extent of conjugation-mediated plasmid transfer during surface-associated growth. We controlled the evaporation-induced hydrodynamic flows within the inoculation droplet by enhancing Marangoni convection (MC) through the addition of a biologically-inert surfactant, conditions that can be self-engineered by bacteria through the production of EPS (extracellular polymeric substances)^39^. In the absence of the surfactant, MC flows are negligible compared to other forces, resulting in a canonical coffee-ring effect (CRE) of the deposited cells. The relative strengths of these two hydrodynamic processes fundamentally alter the local cell density along the initial droplet periphery. The CRE concentrates cells at the initial droplet periphery, which results in a higher number of interspecific contacts (Figs. 2 and 3) and a greater extent of plasmid transfer (Fig. 4) during subsequent surface-associated growth. In contrast, MC results in a more homogeneous distribution of cells across the surface, which results in a lower number of interspecific contacts (Figs. 2 and 3) and a lesser extent of plasmid transfer (Fig. 4) during subsequent surface-associated growth. Importantly, cell deposition by the CRE and MC modulates the density of bacterial cells at the actively growing biomass periphery, which concomitantly impacts subsequent processes during surface-associated growth, such as the persistence of costly mutations that would rapidly disappear in the absence of mechanical forces^40,41^. Thus, evaporation creates hydrodynamic flows that have important consequences on spatial organization and plasmid transfer within surface-associated microbial communities, and is therefore a driver of adaptation, evolution, and functional novelty.

The evaporation-induced hydrodynamic redistribution of cells also governs the prominence of the priority effect (defined as the impact of a species on the overall community establishment due to its prior arrival at a site). When growing on a surface in the absence of competition for nutrients, cells fill the available surface close to the available Voronoi area^42^. A Voronoi partition attributes each point in space to the closest cell in its vicinity, resulting in polygons where the boundaries represent the equidistant lines between two cells. In surface-associated microbial communities, individually-deposited cells approximately fill this available space during growth which results in small, isogenic clusters of kin cells. In comparison to the CRE, MC promotes such clusters of isogenic cells due to the increased average space available around each cell at the initial droplet periphery. Consequentially, many cells behind these microcolonies are restricted from proliferating, which strengthens the priority effect concerning competition for both space and nutrients at the biomass periphery. This effect is also apparent in the simulation results (Fig. 3b). Due to the low intermixing along the initial droplet periphery after cell deposition dominated by MC, there is a slow decrease in intermixing at the biomass periphery. In contrast, after cell deposition dominated by the CRE, there is rapid spatial segregation of strains due to numerous boundary coalescence events (spatial bottlenecks) and loss of individual lineages during surface-associated growth. In the experimental results this process is less evident (Fig. 3a). A key driver of this discrepancy lies in the initial density of bacterial cells at the initial droplet periphery. In the simulations, we simulated cell deposition by the CRE by densely packing cells along a ring of width 1 µm and a radius of 100 µm (i.e., we inoculated a total of 628 cells with no space between individual cells). The consequence is that coalescence events can occur immediately, thus resulting in an immediate decay in intermixing. In the experiments, we inoculated approximately 1000 cells inside a 1 µm droplet. Assuming that the CRE deposits all of these cells along a ring with a radius of 1400 µm (taken from the observed inoculation zone in our experiments; Fig. 2a), this would result in a mean distance of 10 µm between inoculated cells. The greater distances between cells means that coalescence events will not occur immediately, thus resulting in a slower decay in intermixing. We therefore expect a less pronounced decline in the intermixing index for the experiments than for the simulations.

The initial deposition of cells also mediates the proliferation potential of cells having a detrimental or beneficial genetic change within the community. In both our experiments and simulations, carrying a plasmid is detrimental because the plasmid does not provide a beneficial function to the carrier. The cell therefore needs to devote additional resources to replicate the plasmid and express the fluorescent protein. Nevertheless, the plasmid was maintained during surface-associated growth and managed to rapidly increase in abundance due to transfer to proliferating lineages. The survival of slower growing strains due to physical interactions during surface-associated growth has been observed previously^41^. Interestingly, faster growing cells (e.g., those carrying a beneficial genetic change or a plasmid that conveys a competitive advantage) also increase in frequency more slowly than expected in the absence of physical interactions^41^. By concentrating the cells at the initial droplet periphery, a cell carrying a beneficial genetic change has a higher chance of proliferation due to preferential access to both nutrients and space when cell deposition is dominated by the CRE. In contrast, to have a chance of proliferation when cell deposition is dominated by MC, the same cell relies on entirely stochastic processes to be deposited at or near the initial droplet periphery. This pattern is inverted within the inoculation area. Because MC deposits bacterial cells more homogeneously, more transconjugant cells are observed within the inoculation area when compared to cell deposition by the CRE where cells are concentrated at the droplet periphery. Nevertheless, this does not result in a significant increase in relative transconjugant cells even when we consider the inoculation area when calculating the total transconjugant area (Fig. 4).

In addition to primary conjugation (defined as plasmid transfer from a plasmid donor to a potential recipient cell), the spatial positionings of cells will also determine the rate of secondary conjugation (defined as plasmid transfer from a transconjugant to a potential recipient cell)^43^. In our system, we observed significantly more transconjugants when cell deposition was dominated by the CRE than by MC (Fig. 4b). We conclude that the rate of primary conjugation exceeds that of secondary conjugation because the opposite would result in a more equilibrated community concerning the relative abundance of transconjugants. Moreover, rapid secondary conjugation would result in bacterial sectors that are dominated by transconjugants close to the inoculation area (where the total time available for primary and secondary conjugation is longer compared to the biomass periphery) and more potential recipients at the biomass periphery. This is especially relevant for cell deposition dominated by MC, which results in wide sectors of potential recipient cells. However, we do not observe this pattern in our experiment (Fig. 4e), which again supports our conclusion that primary conjugation is the dominant mechanism of plasmid transfer in our system.

Controlling all parameters in the experimental system, such as the exact droplet size and the local cell density at the initial droplet periphery, is nearly impossible. Using our individual-based computational model, we were able to explore the resulting intermixing and plasmid transfer as a function of the total number of inoculated cells, the width of the initial deposition ring, and the overall droplet size (Fig. 5 and Supplementary Figs. 2-4). We found a clear relationship between the initial local cell density and both the intermixing index (Fig. 5b) and the relative transconjugant area (Fig. 5c). These results suggest that beyond natural systems, the abiotic conditions (e.g. temperature, relative humidity, dryness, etc.) during cell deposition and droplet drying can fundamentally alter the emergent patterns of spatial organization during surface-associated growth. Indeed, a similar influence of the local cell density at the initial droplet periphery on spatial intermixing has been observed in silico^42^. When varying the initial droplet radius, we kept the initial local cell density constant by inoculating a cell on each micron of the initial droplet circumference. Nevertheless, we observed a clear relationship between the initial droplet radius and both the intermixing index at the final biomass periphery (Fig. 5d) and the relative transconjugant area (Fig. 5e). We attribute this observation to the difference in competition for unoccupied space at the biomass periphery (Supplementary Fig. 5). When normalized per cell, a large initial droplet radius results in an overall smaller area for progeny to expand into and would result in a more rapid reduction in intermixing when compared to a smaller initial droplet radius. Together, these results suggest that considering the precise experimental procedures is crucial when comparing different surface-associated growth experiments quantitatively.

In this study, we used the simplest case of competing bacterial strains in resource-rich conditions and the transfer of a plasmid that confers a slight fitness cost to eliminate confounding factors and establish causality. Natural microbial communities can consist of numerous different species and putative interactions with multiple limiting resources. Under these conditions, resource-driven structuring results in a rapid loss of slower-growing strains that is accelerated by strong nutrient-limitation^11^. In contrast, positive metabolite-based interactions depend on the close spatial proximity of the strains to circumvent diffusion limitations^44–46^. This suggests that cell deposition by the CRE could benefit multi-species interactions that are mediated by extracellular metabolites with the potential to shift the balance between negative and positive interactions during surface-associated growth. Finally, natural communities rarely experience prolonged stable conditions. For example, when growing on surfaces, they can experience drying-rewetting cycles that are further associated with dynamic nutrient and oxygen availability. Under these conditions, the temporal dynamics of pattern change and plasmid transfer reported here could be of special importance in driving the diversity of the community as a whole and the spread of deleterious genetic changes. Unraveling these interactions mechanistically is paramount to link the environmentally widely occurring physical processes as encountered in natural sessile populations with the wealth of knowledge gained from controlled laboratory-based experiments of surface-associated growth.

## Methods

### Bacterial strains and culture conditions

We performed all experiments using isogenic mutant strains of the bacterium *E. coli* MG1655. Strains TB204 and TB205 are derivatives of strain MG1655 that constitutively express green and red fluorescent protein, respectively, from the lambda promoter located on the chromosome^47^. We introduced the self-transmissible conjugative plasmid R388, which encodes for cyan fluorescent protein and chloramphenicol resistance^48^, into *E. coli* TB204 by conjugation from *E. coli* DH5α (transconjugants are referred to as strain TB206) using conventional filter mating on agar plates. The average copy number of plasmid R388 is approximately 4^49^. For the plasmid transfer experiments, we used strain TB206 as the R388 donor strain and TB205 as the potential recipient strain. We routinely grew all strains in liquid lysogeny broth (LB) medium at 37°C with shaking. When growing the R388 donor strain in isolation, we supplemented the LB medium with 25 μg/mL chloramphenicol to prevent the proliferation of R388 segregants. For long-term storage, we archived all the strains at −80°C in 15% (v/v) glycerol stocks. For all experiments, we first cultured each strain separately overnight using a single isolated colony obtained from streaking the appropriate -80°C stock onto an LB agar plate.

### Surface-associated growth experiments

We prepared LB agar plates for droplet evaporation from their surfaces by adding 25 g/L LB and 10 g/L bacteriology-grade agar powder (AppliChem, Darmstadt, Germany) to 1000 mL distilled water and autoclaving the medium at 121 °C for 20 min. After cooling to approximately 70 °C, we dispensed 10 mL aliquots of the medium into sterile 3.5 cm diameter petri dishes and let them solidify at room temperature for 2 h. Once solidified, we transferred the agar plates to a sterile laminar flow hood and dried them for 10 min with the lid open. We then covered the agar plates with their lids, sealed them with Parafilm (Amcor, Zürich, Switzerland), and stored them at 4 °C prior to further use.

For the experiments, we first grew individual overnight cultures of the potential recipient strains TB204 (constitutively expressing green fluorescent protein from the chromosome) and TB205 (constitutively expressing red fluorescent protein from the chromosome) and the R388 donor strain TB206 (constitutively expressing green fluorescent protein from the chromosome and cyan fluorescent protein from R388). We then transferred the individual overnight cultures to fresh LB medium at a dilution of 1:100 (vol:vol) and grew them for 4 h at 37 °C with shaking to ensure that the cultures were in the logarithmic growth phase. We next adjusted each culture to an optical density at 600 nm (OD_600_) of 2 in a 1 mL volume (approximately 10^9^ colony forming units [CFU]/mL), washed the cells three times by centrifuging them at 3600 × *g* and 4 °C for 10 min, and resuspended them in 1 mL phosphate buffered saline (PBS). After washing, we diluted the bacterial mixtures to 10^6^ CFU/mL in PBS.

For the surface-associated growth experiments, we induced the CRE or MC using established methods^28^. Briefly, to induce the CRE, we directly used the bacterial suspensions diluted in PBS to initiate the experiments. To promote MC, we added 1% polyethylene glycol (PEG) to the bacterial suspensions, which is a non-toxic and non-metabolizable (biologically inert) surfactant that does not affect the growth of our strains under our experimental conditions (Supplementary Fig. 1a). We mixed equal volumes of the bacterial suspensions (with or without PEG) to obtain the desired strain combinations as indicated in the Results section and deposited 1 μL aliquots of the mixtures onto the centers of separate replicated LB agar plates, where each 1 µl aliquot contained approximately 1000 bacterial cells. Note that 1 µl is within the normal size range of atmospheric precipitation droplets^50^. Moreover, contact of larger droplets with surfaces can (i) disintegrate the droplet into smaller droplets that are on the order of 1 µl, and (ii) transfer up to 0.01% of bacteria from the soil surface^51^. We used strains TB204 and TB205 to test how the CRE and MC affect spatial intermixing during surface-associated growth and strains TB205 (potential recipient) and TB206 (R388 donor) to test how the CRE and MC affect R388 transfer during surface-associated growth. We next transferred the LB agar plates into a humidity-controlled environment (30% relative humidity) for 10 min at room temperature for droplet evaporation and then incubated the LB agar plates at 21 °C for 4 days, which allowed for sufficient surface-associated growth for us to quantify experimentally. We performed five independent experimental replicates for each treatment.

### Microscopy and image analysis

We imaged the resulting microbial biomass after surface-associated growth using a Leica TCS SP5 II CLSM (Leica Microsystems, Wetzlar, Germany) equipped with a 5× HCX FL objective, a numerical aperture of 0.12, and a frame size of 1024 × 1024 (resulting in a pixel size of 3.027 µm). We used a laser emission of 458 nm for the excitation of cyan fluorescent protein, 488 nm for the excitation of the green fluorescent protein, and 514 nm for the excitation of the red fluorescent protein. We set the emission filter to 469–489 nm for cyan fluorescent protein, 519–551 nm for green fluorescent protein, and 601–650 nm for red fluorescent protein. We quantified the relative transconjugant area by measuring the area occupied by cells that express both cyan and red fluorescent protein (i.e., where the recipient carries R388) and dividing it by the total biomass area, which is equivalent to the area occupied by cells that express red or green fluorescent protein.

We used the spatial intermixing index as a proxy measure of the number of contacts between different strains^44,52^. Briefly, we drew a circle positioned at a defined radial distance from the initial droplet periphery and quantified the number of color transitions (each strain expresses a different fluorescent protein) along the circle. We then normalized the number of color transitions by the circumference of the circle^44,52^. We repeated this quantification for different radii to obtain intermixing as a function of the extent of radial growth. To achieve this, we used Fiji (v. 2.1.0/1.53c) (https://fiji.sc)^44,53^. Briefly, we used the Fiji default algorithm to threshold and binarize each image and the built-in ‘remove outliers’ method (radius = 5, threshold = 50, bright) to remove noise. We then used the Sholl plugin^54^ of ImageJ (https://imagej.net) to quantify the number of transitions between background and information containing parts of the image using a concentric windowing approach from the initial droplet periphery to the final biomass periphery at the end of the experiment at radial increments of 5 µm. We then calculated the intermixing index *I*_*r*_ as the number of color transitions *N*_*r*_ divided by the expected number of color transitions for a random spatial distribution of two strains at a certain radius *r* as shown in Eq. 1 and described elsewhere^44^.

1 $$I_r = \frac{{N_r}}{{\pi ^r/2}}$$

### Individual-based computational modeling

We used CellModeller 4.3^55^ as a modular platform for individual-based computational modelling to gain further insights into our experimental results and systematically examine initial cell deposition patterns that we could not test experimentally. We simulated surface-associated cell growth across a two-dimensional plane (bacterial monolayer) where the inoculum contained a 1:1 mixture (cell number:cell number) of two strains (colored red or green but were otherwise phenotypically identical) that were homogeneously deposited and randomly rotated across a defined space to start the simulation. We used a circular ring with a specific ring width as the initial cell deposition area (inoculation area). Using this approach, we simulated the relative strength of the CRE and MC by changing the width of the circular ring (see Fig. 5a). In the extreme case when the ring width equaled the droplet radius, the system was dominated by MC (i.e. homogeneous deposition of cells across the entire droplet area). When the ring width was small compared to the overall droplet radius, the system was dominated by the CRE (i.e., concentration of cells at the droplet periphery). We deposited the cells randomly across the inoculation area and assigned an initial rotation of each rod-shaped cell at random to start the simulation. Using this approach, we could quantitatively assess the effects of different evaporation-induced initial cell deposition patterns by systematically varying the droplet radius, the circular ring width, and the number of deposited cells.

We simulated rod-shaped cells of radius 0.5 µm and length of 2 µm to represent *E. coli* cells. Cells divided when they reached the length of a random value set between 3.5 and 4.0 µm. We set the cell growth rate to 2, which means that cells grew 2 µm in length per unit time step. Populations expanded across space following physically-resolved cell shoving based on cell-cell contact and modelled with the ‘collision detection and collision response’ module included in CellModeller^55^. In this study, we simulated the plasmid by changing the specific growth rate of the plasmid donor strain to 1.9 (from 2) to account for a plasmid-associated maintenance cost. Because we did not use any antibiotics or apply any positive selection for the plasmid, there was no benefit of carrying the plasmid. We included plasmid transfer by detecting cell-cell contacts between strains using the CompNeighbours function in CellModeller^55^ and applying a constant probability of 0.001 for successful plasmid transfer upon contact. If successful, we assigned the transconjugant cell a different color (blue) and set the specific growth rate to 1.9. We summarized the parameters that we used in Supplementary Table 3 and provided a list of simulation conditions in Supplementary Table 1. Because all parameter values are relative in the model, we selected the parameters such that the simulation results qualitatively matched the experimental observations. We stored the status and spatial location of each cell every 20 time-steps and we performed simulations until completing 560 time-steps. Due to computational limitations, the number of cells in the simulations was approximately 10% of the number of cells in the experiments, but this had no effect on the mechanical/physical interactions that occurred between cells or on the qualitative patterns of spatial organization that we observed. We performed five independent simulation replicates for each condition.

### Statistical analyses

We performed all statistical tests in R (v. 4.1.2) (https://cran.r-project.org). We assessed statistical significance between means of the CRE and MC factor levels using two-sample two-sided Welch tests. We therefore did not make any assumptions regarding the homoscedasticity of our datasets. We corrected all *p*-values for multiple comparisons using the Holm-Bonferroni method. To describe trends in spatial intermixing at the biomass periphery, we performed linear regressions between spatial intermixing and the extent of radial growth for each replicate and extracted the slopes. We then compared the mean slopes between treatment levels using two-sample two-sided Welch tests with the Holm-Bonferroni correction as described above. We used the Wilk-Shapiro test to test the normality of our datasets and considered *p* > 0.05 to validate the assumption of normality (Supplementary Table 4). We reported the statistical test, *p*-value, and sample size for each test in the Results section.

### Reporting summary

Further information on research design is available in the Nature Research Reporting Summary linked to this article.

### Supplementary information

Supplementary Information

Reporting Summary

Supplementary Movie 1

Supplementary Movie 2

Supplementary Movie 3

Supplementary Movie 4

## Data Availability

All data generated in this study are publicly available in the Eawag Research Data Institutional Collection (ERIC) repository (https://opendata.eawag.ch/) at the following 10.25678/0007JM.

## Code availability

All codes used in this study are publicly available in the Eawag Research Data Institutional Collection (ERIC) repository (https://opendata.eawag.ch/) at the following 10.25678/0007JM.
