# Supplementary material for: Evaporation-induced hydrodynamics control plasmid transfer during surface-associated microbial growth
Source: NPJ Biofilms Microbiomes. 2023 Aug 22;9:58. doi: 10.1038/s41522-023-00428-x (PMC10444754; doi:10.1038/s41522-023-00428-x)
Supplement: Supplementary file 1 — Supplementary Information [file 41522_2023_428_MOESM1_ESM.pdf]

SUPPLEMENTARY INFORMATION

Evaporation-induced hydrodynamics control plasmid transfer during surface-associated microbial growth

Chujin Ruan[1,2‡], Benedict Borer[3‡], Josep Ramoneda[2,4], Gang Wang[1,5] & David R. Johnson[2,6].

[1]College of Land Science and Technology, China Agricultural University, 100193 Beijing, China; [2]Department of Environmental Microbiology, Swiss Federal Institute of Aquatic Science and Technology (Eawag), 8600 Dübendorf, Switzerland; [3]Department of Earth, Atmospheric and Planetary Sciences, Massachusetts Institute of Technology, Cambridge, MA 02139, USA; [4]Cooperative Institute for Research in Environmental Sciences, University of Colorado, Boulder, CO 80309, USA; [5]National Black Soil & Agriculture Research, China Agricultural University, 100193 Beijing, China; [6]Institute of Ecology and Evolution, University of Bern, 3012 Bern, Switzerland. [‡]These authors contributed equally to this work.

Correspondence:
David R. Johnson, david.johnson@eawag.ch
Gang Wang, gangwang@cau.edu.cn

This file includes:
Supplementary Tables 1-4
Supplementary Figures 1-5

Supplementary Table 1: Initial conditions for individual-based computational simulations of surface-associated growth.

Variable/parameter description	Units	Value
**Cell deposition dominated by MC**		
Ring width	µm	100
Initial droplet radius	µm	100
Initial cell number	# cells	628
**Cell deposition dominated by the CRE**		
Ring width	µm	1
Initial droplet radius	µm	100
Initial cell number	# cells	628
**Ring density variation (CRE)**		
Ring width	µm	1
Initial droplet radius	µm	100
Ring density 1	# cells	628
Ring density 0.8	# cells	502
Ring density 0.6	# cells	378
Ring density 0.4	# cells	250
Ring density 0.2	# cells	126
**Initial droplet radius variation (CRE)**		
Ring width	µm	1
Ring density	-	1
Initial droplet radius 30 µm	# cells	188
Initial droplet radius 60 µm	# cells	376
Initial droplet radius 90 µm	# cells	566
Initial droplet radius 120 µm	# cells	754
Initial droplet radius 150 µm	# cells	942
**Ring width variation (CRE)**		
Initial droplet radius	µm	100
Ring density	-	1
Ring width 1 µm	# cells	628
Ring width 10 µm	# cells	628
Ring width 20 µm	# cells	628
Ring width 30 µm	# cells	628
Ring width 40 µm	# cells	628

Supplementary Table 2: Statistics and coefficients of linear regressions in Figure 5.

	**Intercept**	**Slope**	**R^2**	***Pearson's R***
Intermixing index versus cell density (panel A)	0.062	0.052	0.35	0.59
Transconjugant area versus cell density (panel B)	0.051	0.050	0.82	0.91
Intermixing index versus droplet radius (panel C)	0.134	$-3.22*10^{-4}$	0.25	-0.50
Transconjugant area versus droplet radius (panel D)	0.112	$-1.64*10^{-4}$	0.56	-0.76

Supplementary Table 3: Model parameters used for individual-based computational simulations of surface-associated growth.

Parameter	Description	Value	Unit
g_n	Standard specific growth rate	2	-
$g_{plasimd}$	Specific growth rate when carrying a plasmid	1.9	-
L_0	Mean initial cell length	2	µm
L_d	Mean cell length at division	3.5	µm
R	Radius of the inoculation area	100	µm
d_{cell}	Mean diameter of a cell	0.5	µm
P	Probability of plasmid transfer	0.001	-

Supplementary Table 4: Tests of normality of the data sets reported in the study.

		Shapiro-Wilk test	
**Dataset**	**n**	**W**	***P***
Coffee ring effect on number of sectors (experimental)	5	0.949	0.726
Marangoni effect on number of sectors (experimental)	5	0.917	0.512
Coffee ring effect on number of sectors (simulation)	5	0.964	0.833
Marangoni effect on number of sectors (simulation)	5	0.951	0.745
Coffee ring effect on intermixing (experimental)	5	0.943	0.688
Marangoni effect on intermixing (experimental)	5	0.908	0.456
Coffee ring effect on intermixing (simulation)	5	0.966	0.847
Marangoni effect on intermixing (simulation)	5	0.938	0.651
Coffee ring effect on plasmid transfer (experimental)	5	0.870	0.268
Marangoni effect on plasmid transfer (experimental)	5	0.894	0.380
Coffee ring effect on plasmid transfer (simulation)	5	0.895	0.385
Marangoni effect on plasmid transfer (simulation)	5	0.985	0.958
Coffee ring effect on total cell counts (experiment)	5	0.955	0.774
Marangoni effect on total cell counts (experiment)	5	0.896	0.390
Ring width, radius and density effects on intermixing (simulation)	5	0.975	0.154
Ring width, radius and density effects on plasmid transfer (simulation)	5	0.941	0.002

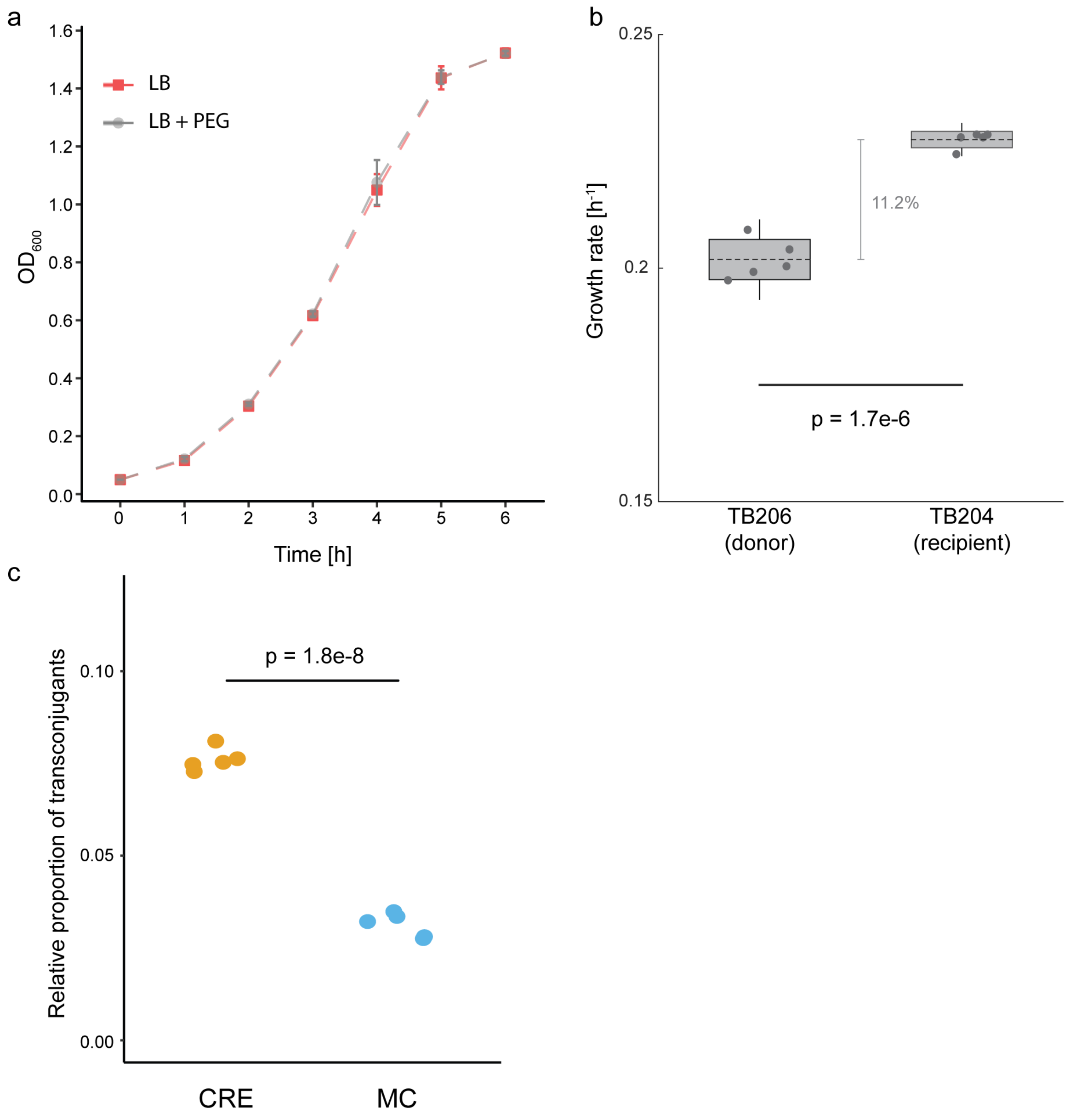

Supplementary Fig. 1: Influence of polyethylene glycol and plasmid presence on the growth of *E. coli* and quantification of transconjugants using selective plate counting. a Strain *E. coli* TB205 was incubated in a 37°C shaking incubator for 6 hours in LB medium. Three experimental replicates were performed for each condition. Data points are means and error bars are standard deviations of the experimental replicates. **b** Quantification of growth rates of the R388 plasmid donor (strain TB206) and the potential recipient (strain TB204). Carrying R388 results in a reduction in the growth rate by ~11%. The *p*-value is for a two-sample two-sided Welch test. **c** Quantification of the total number of transconjugants in each range expansion by selective plating on LB agar plates amended with 30 µg/mL chloramphenicol. Five experimental replicates were performed for each condition. Each datapoint is the measurement for a separate experimental replicate. The *p*-value is for a two-sample two-sided Welch test.

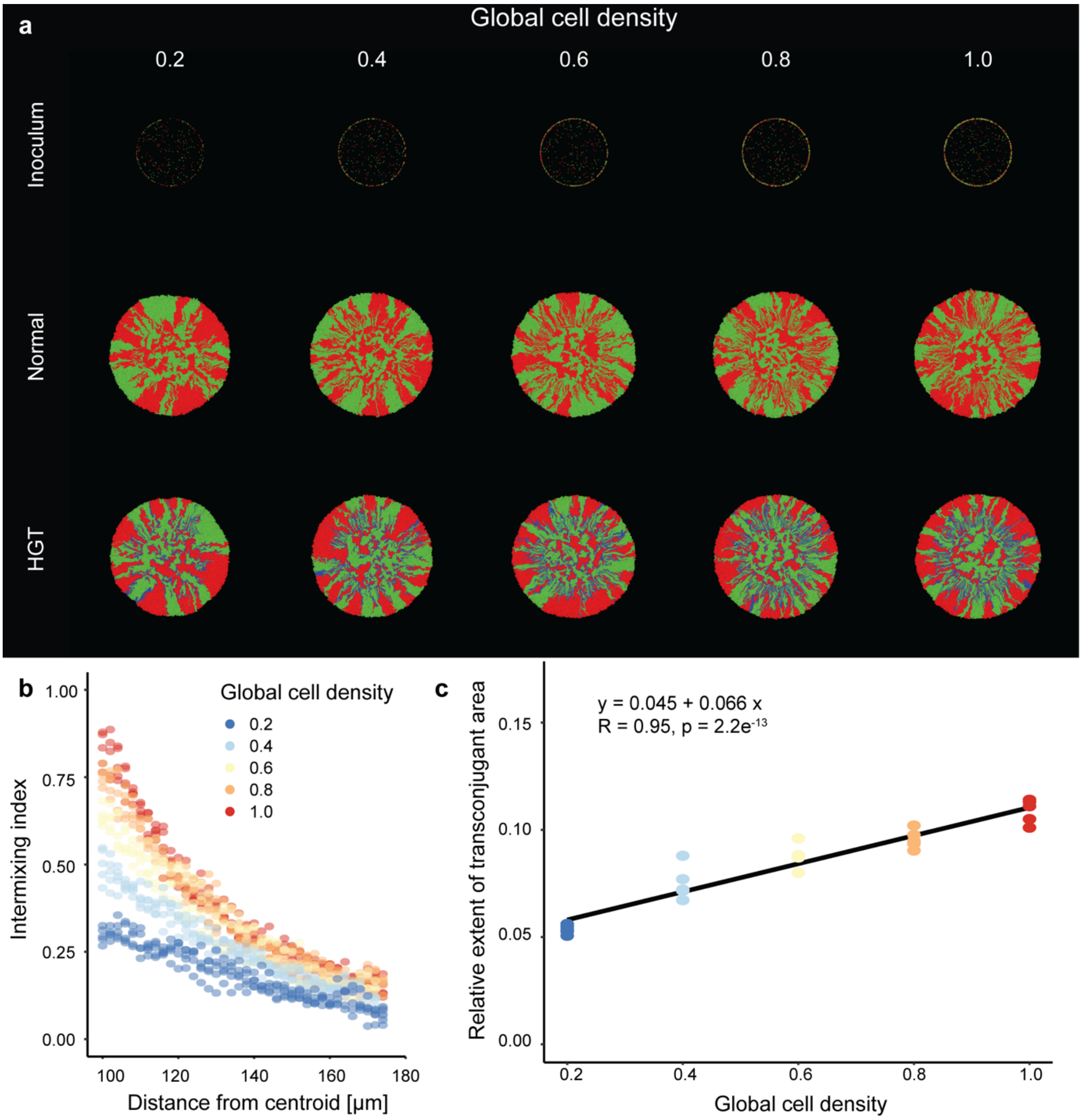

Supplementary Fig. 2: Influence of the total inoculated cell number on spatial intermixing and plasmid transfer. Note that higher total inoculated cell numbers correspond to higher local cell densities at the initial droplet periphery (ring density). **a** Representative simulations of the inoculum (Inoculum), of surface-associated growth for two competing strains in the absence of a plasmid (Normal), and of surface-associated growth for two competing strains in the presence of a conjugative plasmid (HGT; transconjugants are blue). **b** Quantification of spatial intermixing as a function of the distance from the centroid for two competing strains with different total inoculated cell numbers (Normal). **c** Relative transconjugant area for two competing strains with different total inoculated cell numbers (HGT). For **b** and **c**, each datapoint is a measurement for one of five simulation replicates. For **c**, the line is a linear regression fit to the data.

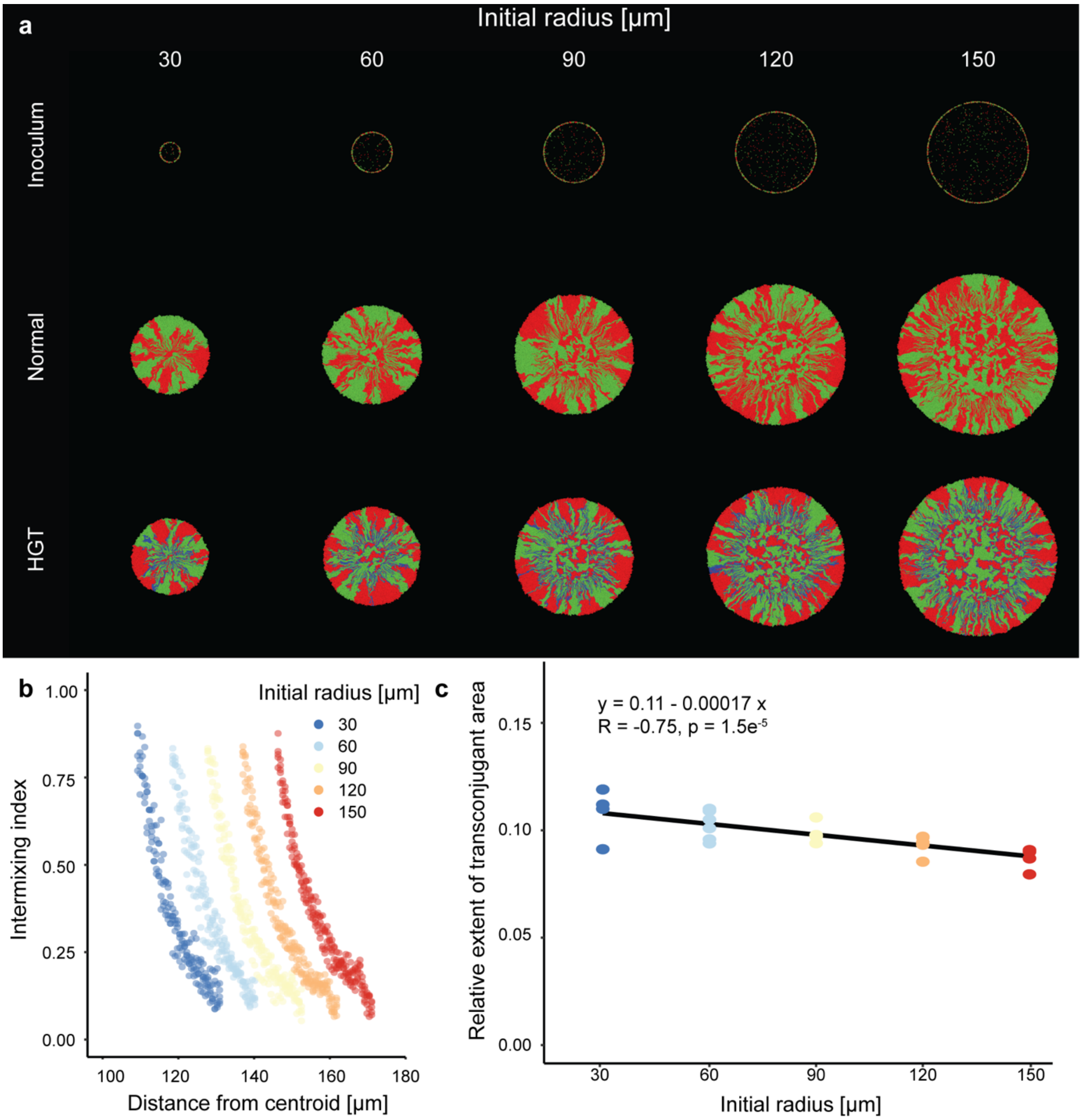

Supplementary Fig. 3: Influence of the initial simulated droplet radius on spatial intermixing and plasmid transfer. **a** Representative simulations of the inoculum (Inoculum), of surface-associated growth for two competing strains in the absence of a plasmid (Normal), and of surface-associated growth for two competing strains in the presence of a conjugative plasmid (HGT; transconjugants are blue). **b** Quantification of spatial intermixing as a function of the distance from the centroid for two competing strains with different initial droplet radii (Normal). **c** Relative transconjugant area for two competing strains with different initial droplet radii (HGT). For **b** and **c**, each datapoint is a measurement for one of five simulation replicates. For **c**, the line is a linear regression fit to the data.

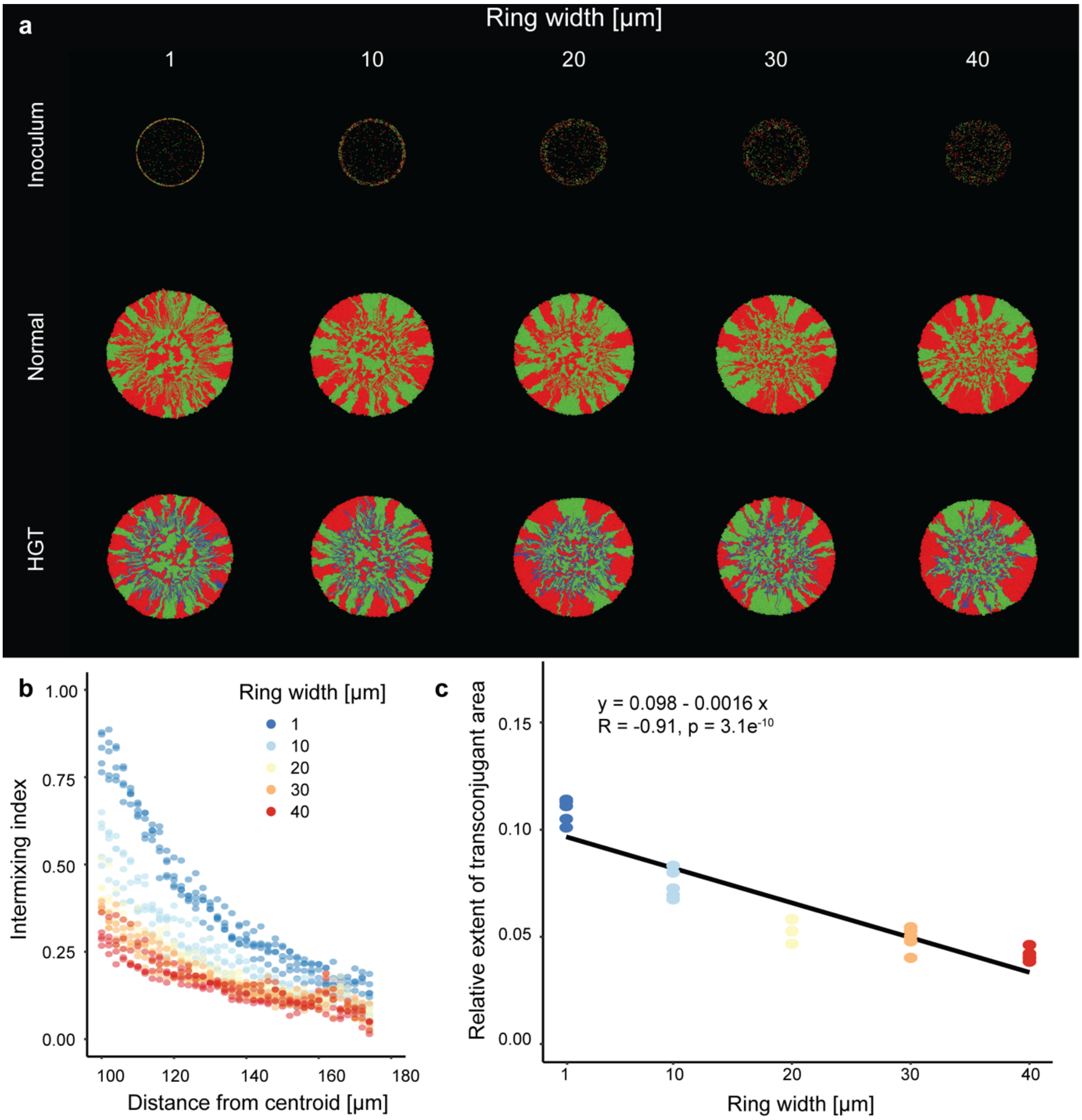

Supplementary Fig. 4: Influence of the inoculum ring width on spatial intermixing and plasmid transfer. **a** Representative simulations of the inoculum (Inoculum), of surface-associated growth for two competing strains in the absence of a plasmid (Normal), and of surface-associated growth for two competing strains in the presence of a conjugative plasmid (HGT; transconjugants are blue). **b** Quantification of spatial intermixing as a function of the distance from the centroid for two competing strains with different ring widths (Normal). **c** Relative transconjugant area for two competing strains with different ring widths (HGT). For **b** and **c**, each datapoint is a measurement for one of five simulation replicates. For **c**, the line is a linear regression fit to the data.

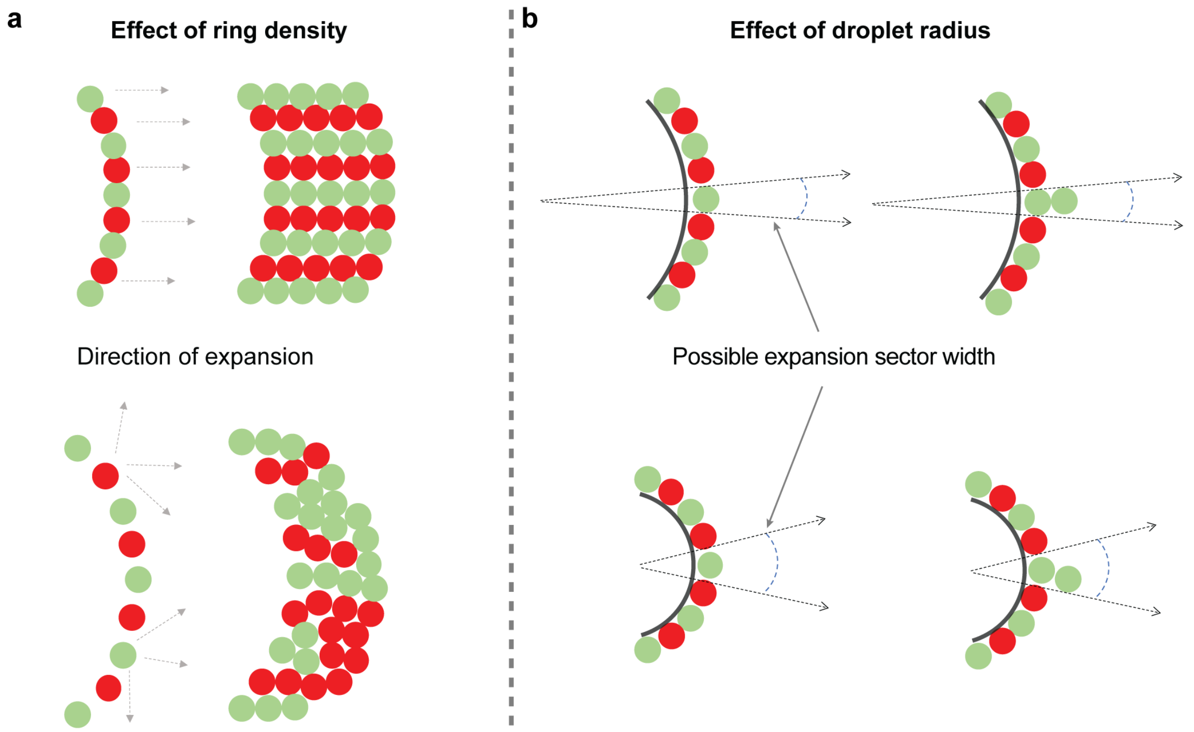

Supplementary Fig. 5: Influence of the local cell density at the initial droplet periphery (ring density) and droplet radius on competition for unoccupied space. a The ring density on a surface determines the available space for proliferation of cells. In a high-density scenario, each cell has limited space to place a daughter cell upon cell division, leading to a structured pattern of highly intermixed patches. By contrast, in a low-density scenario, each cell has more space to place a daughter cell, leading to the stochastic formation of irregular and clustered patches. **b** Similarly, the initial droplet radius also determines the possible sector width during surface-associated growth. Larger droplet radii lead to a narrower cell placement angle whereas smaller droplet radii lead to a larger cell placement angle.
